# Solitary Testicular Metastasis as the Only Site of Recurrence in Prostate Adenocarcinoma

**DOI:** 10.7759/cureus.115220

**Published:** 2026-08-26

**Authors:** Mohamed Amine Harchaoui, Brahim Masaoudi, Mustapha Azzakhmam, Mohammed Alami, Ahmed Ameur

**Affiliations:** 1 Urology, Mohammed V Military Instruction Hospital, Rabat, MAR; 2 Pathology, Mohammed V Military Instruction Hospital, Rabat, MAR

**Keywords:** immunohistochemistry markers, oligometastatic prostate cancer, prostate adenocarcinoma, psma pet-ct, solitary testicular metastasis

## Abstract

Prostate cancer preferentially metastasizes to bone and lymph nodes. Testicular metastases are exceptionally rare and are typically observed in the setting of advanced polymetastatic disease. Their presentation as an isolated oligometastatic relapse remains highly unusual.

We report the case of a 76-year-old patient followed since 2019 for de novo metastatic prostate adenocarcinoma, initially treated with androgen deprivation therapy (ADT) with a complete response, who presented in March 2026 with a left testicular mass. Germ cell tumor markers were negative, while prostate-specific antigen (PSA) was elevated at 30 ng/mL. Prostate-specific membrane antigen positron emission tomography-computed tomography (PSMA PET-CT) demonstrated increased uptake involving both the testis and the peri-scrotal region. A radical inguinal orchiectomy was performed. Immunohistochemical analysis showed strong expression of prostein (P501S) and alpha-methylacyl-CoA racemase (AMACR), confirming the prostatic origin of the lesion.

Postoperatively, systemic treatment was intensified using combined androgen deprivation therapy with abiraterone acetate, resulting in a marked decline in PSA levels. Testicular dissemination of prostate adenocarcinoma may occur through retrograde venous or lymphatic pathways. Diagnosis relies on advanced molecular imaging and immunohistochemistry, particularly in cases with reduced PSA expression. This case highlights the relevance of a multimodal strategy in oligometastatic disease, combining metastasis-directed surgery and intensified systemic therapy.

## Introduction

Prostate cancer is one of the most common malignant neoplasms in the global male population [1]. In the vast majority of cases, the natural history of advanced disease is characterized by metastatic dissemination with a particularly well-documented tissue tropism. Prostate tumor cells demonstrate a pronounced organ-specific affinity for the bone microenvironment and locoregional and distant lymphatic basins. Large-scale epidemiological studies and extensive autopsy series reveal that in advanced stages of the disease, secondary localizations are predominantly found in the skeleton (approximately 84% to 90% of cases), followed by distant lymph nodes (approximately 10.6%), the lungs (between 9.1% and 46%), and the liver (approximately 25%) [2]. Contrasting with this classic distribution, metastatic involvement of the testicular parenchyma, originating from a primary prostate adenocarcinoma, emerges as an exceptionally rare clinical and pathological entity. While testicular metastases are incidentally discovered in approximately 2% to 4% of autopsy series in patients with advanced prostate cancer, clinically recognized cases during a patient's lifetime remain exceedingly rare [3]. We report here the exceptional case of a patient who developed an isolated unilateral testicular metastasis from a prostate adenocarcinoma, seven years after the initial diagnosis and while under hormonal treatment.

## Case presentation

We report the case of a 76-year-old male patient with a medical history of arterial hypertension. This patient has been followed since 2019 for a de novo metastatic prostate adenocarcinoma, defined as high-volume according to the CHAARTED criteria (Gleason score 8 [4+4]) [1]. At initial diagnosis, conventional staging using a computed tomography (CT) scan of the chest, abdomen, and pelvis, alongside bone scintigraphy, revealed multiple secondary bone and lymph node lesions. Systemic treatment with androgen deprivation therapy (ADT) coupled with an androgen receptor pathway inhibitor (ARPI) (abiraterone acetate) was initiated upon diagnosis, yielding an excellent clinical and biological response. Indeed, six months after treatment initiation, the prostate-specific antigen (PSA) level dropped from an initial value of 150 ng/mL to a nadir of 0.08 ng/mL, accompanied concurrently by the complete disappearance of active secondary lesions on follow-up imaging.

**Figure 1:**
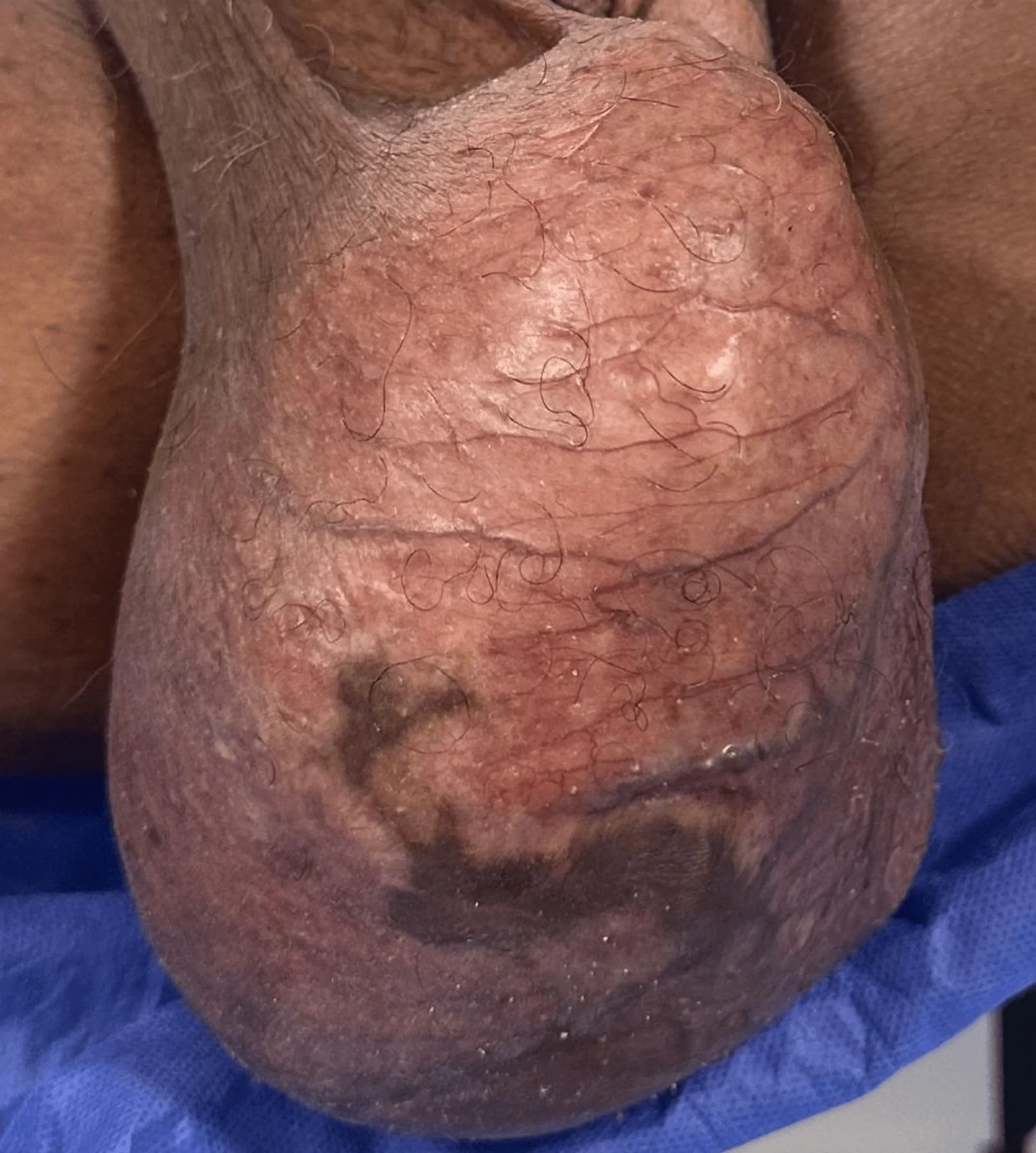
Preoperative clinical photograph of the left hemiscrotum. This clinical image, taken immediately prior to surgical intervention, illustrates a massive, asymmetrical, and global enlargement of the left hemiscrotum. The overlying scrotal skin remains intact and is devoid of overt inflammatory signs, such as acute erythema or edema. This macroscopic presentation corresponds directly to the patient's clinical complaint of a painless swelling, driven by the underlying solid intratesticular metastasis and the associated reactive hydrocele.

After being lost to follow-up during 2025, the patient presented again in March 2026 with a global and painless increase in scrotal volume. The initial physical examination revealed a left testicular mass of firm consistency, painless to palpation, with no overlying inflammatory signs (Figure 1).

Scrotal ultrasound demonstrated significant heterogeneous and nodular alterations of the left testicular parenchyma, associated with a reactive hydrocele.

**Figure 2:**
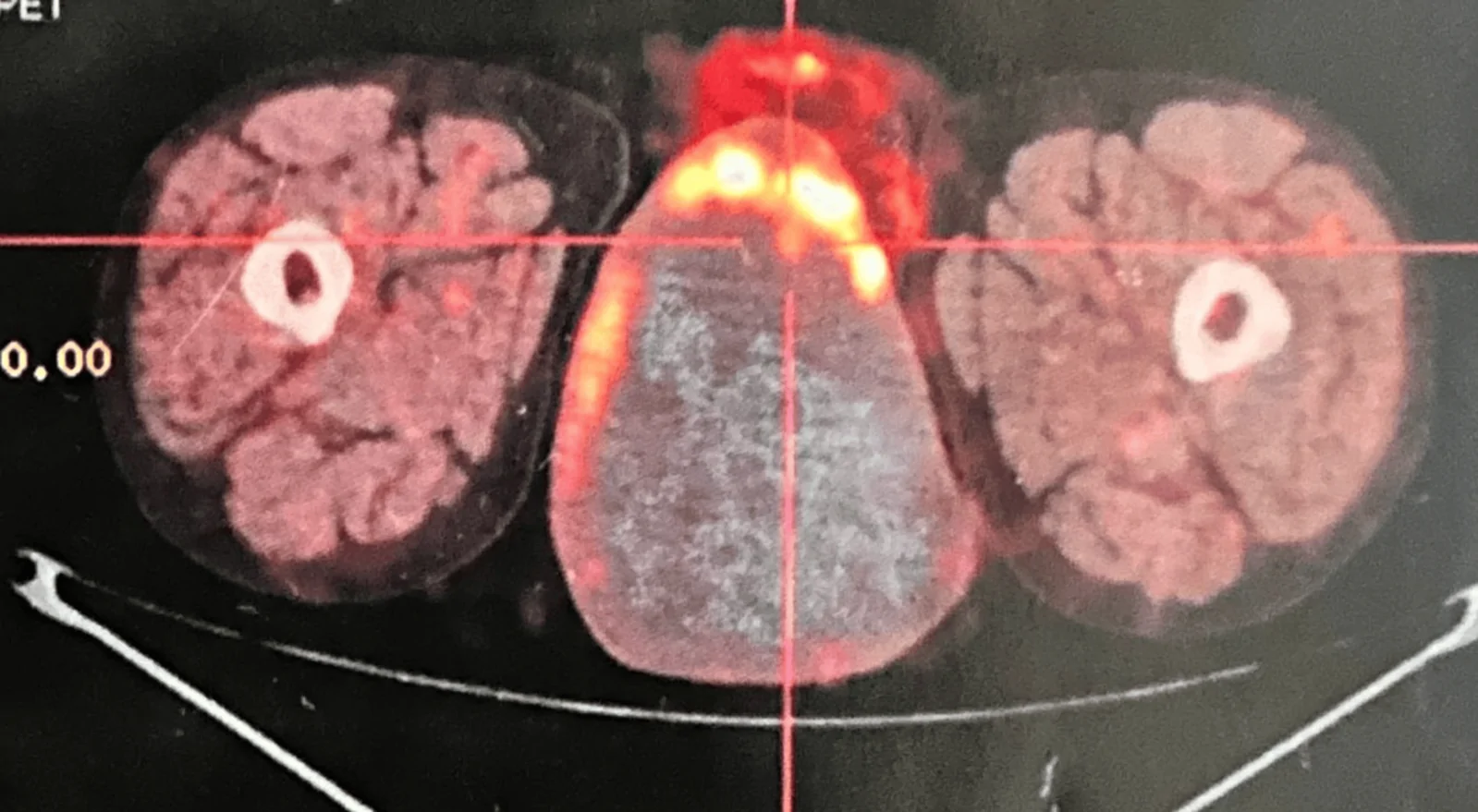
Axial prostate-specific membrane antigen (PSMA) PET-CT revealing a solitary, intensely hypermetabolic left intratesticular focus (SUVmax 18).

Biologically, the evaluation of testicular tumor markers (beta-human chorionic gonadotropin (β-hCG) and alpha-fetoprotein (AFP)) was negative. A slight increase in lactate dehydrogenase (LDH) was noted. Concomitantly, the serum PSA level was elevated to 30 ng/mL. A prostate-specific membrane antigen positron emission tomography-computed tomography (PSMA PET-CT) was performed. The examination revealed an intense pathological left intratesticular hypermetabolic focus (maximum standardized uptake value (SUVmax) of 18), associated with circumferential periscrotal uptake and a non-avid hydrocele (Figure 2). The whole-body scan found no other distant metastatic localizations.

**Figure 3:**
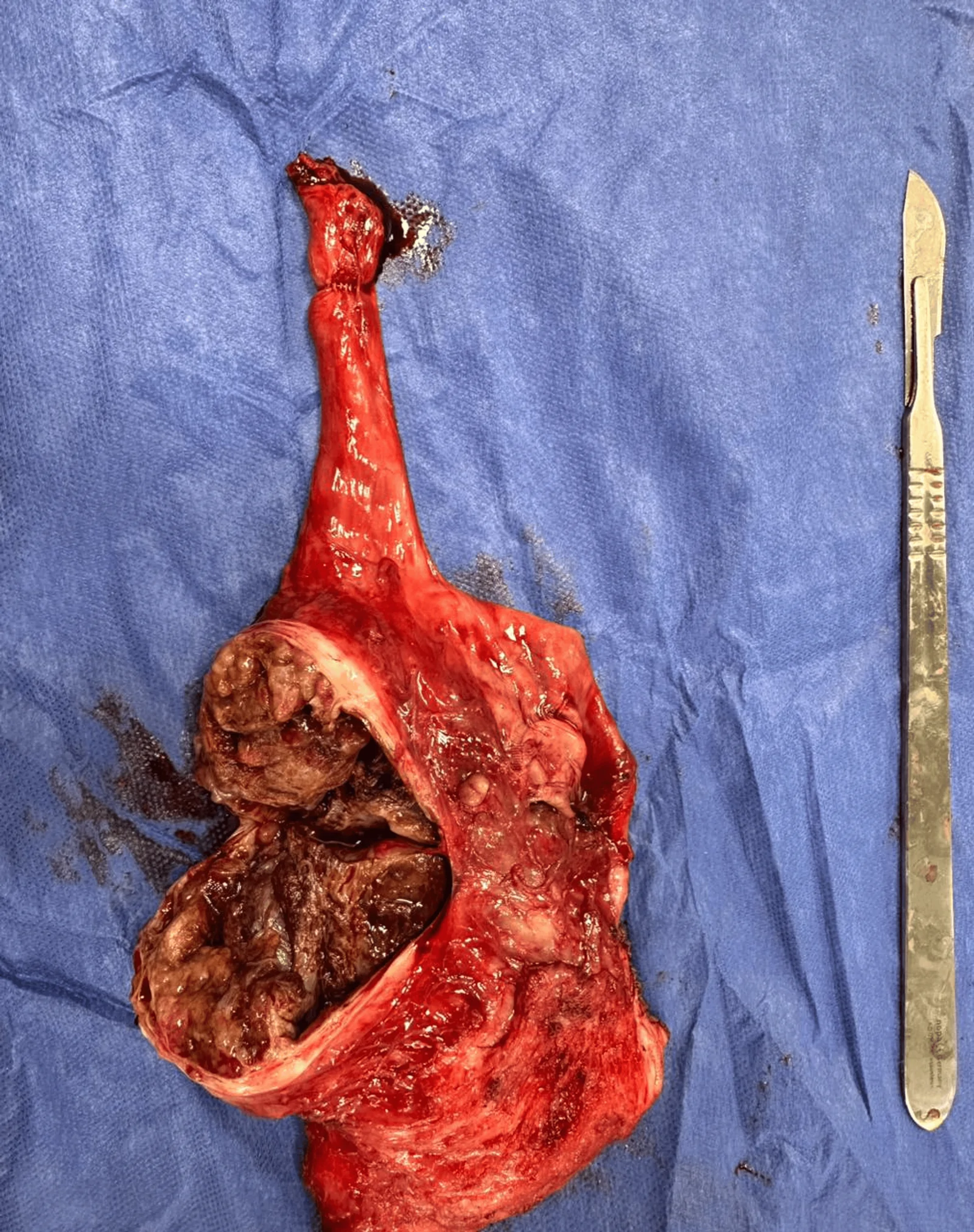
Macroscopic specimen of the bisected left radical inguinal orchiectomy following drainage of the reactive hydrocele, revealing a massive, hemorrhagic tumor replacing the native testicular parenchyma.

The patient underwent surgical management consisting of a left radical inguinal orchiectomy (Figure 3).

**Figure 4:**
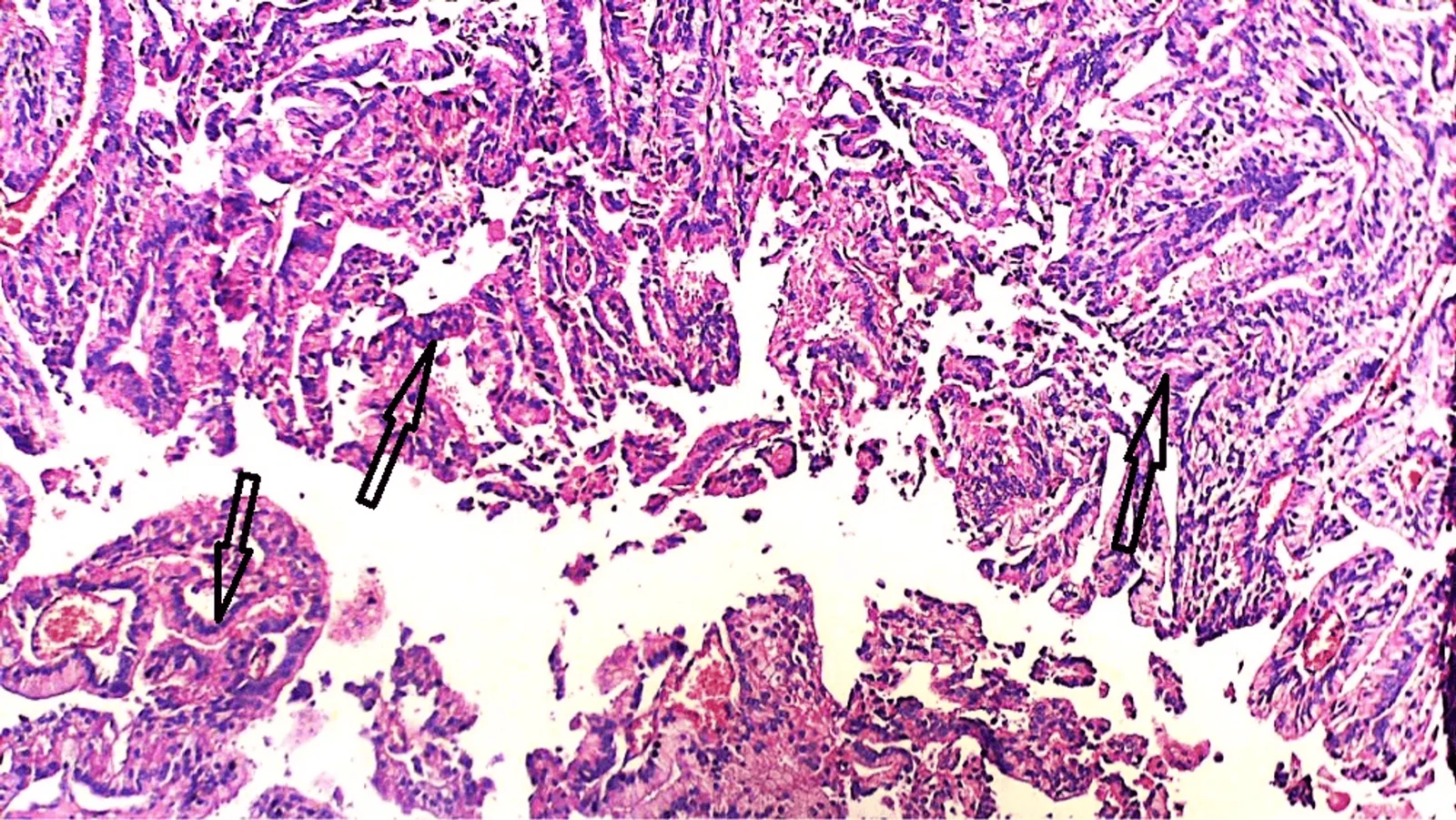
Histopathological features of the testicular tumoral proliferation (Hematoxylin and Eosin - H&E stain) Medium power (x20). Black arrows indicating tumoral proliferation made of atypical glands.

**Figure 5:**
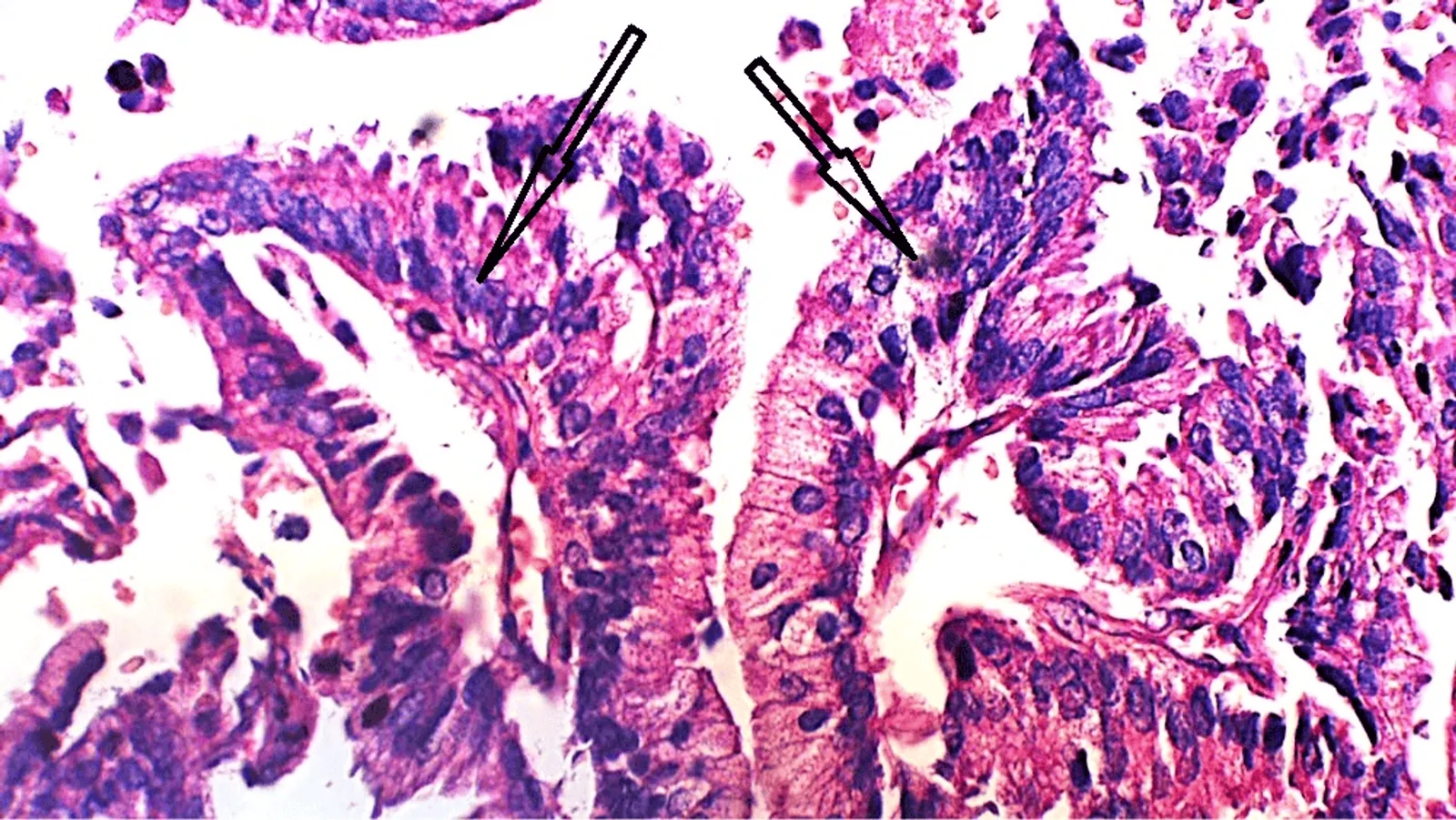
Histopathological features of the testicular tumoral proliferation (Hematoxylin and Eosin - H&E stain) Medium power (x40). Black arrows indicating tumoral cells.

**Figure 6:**
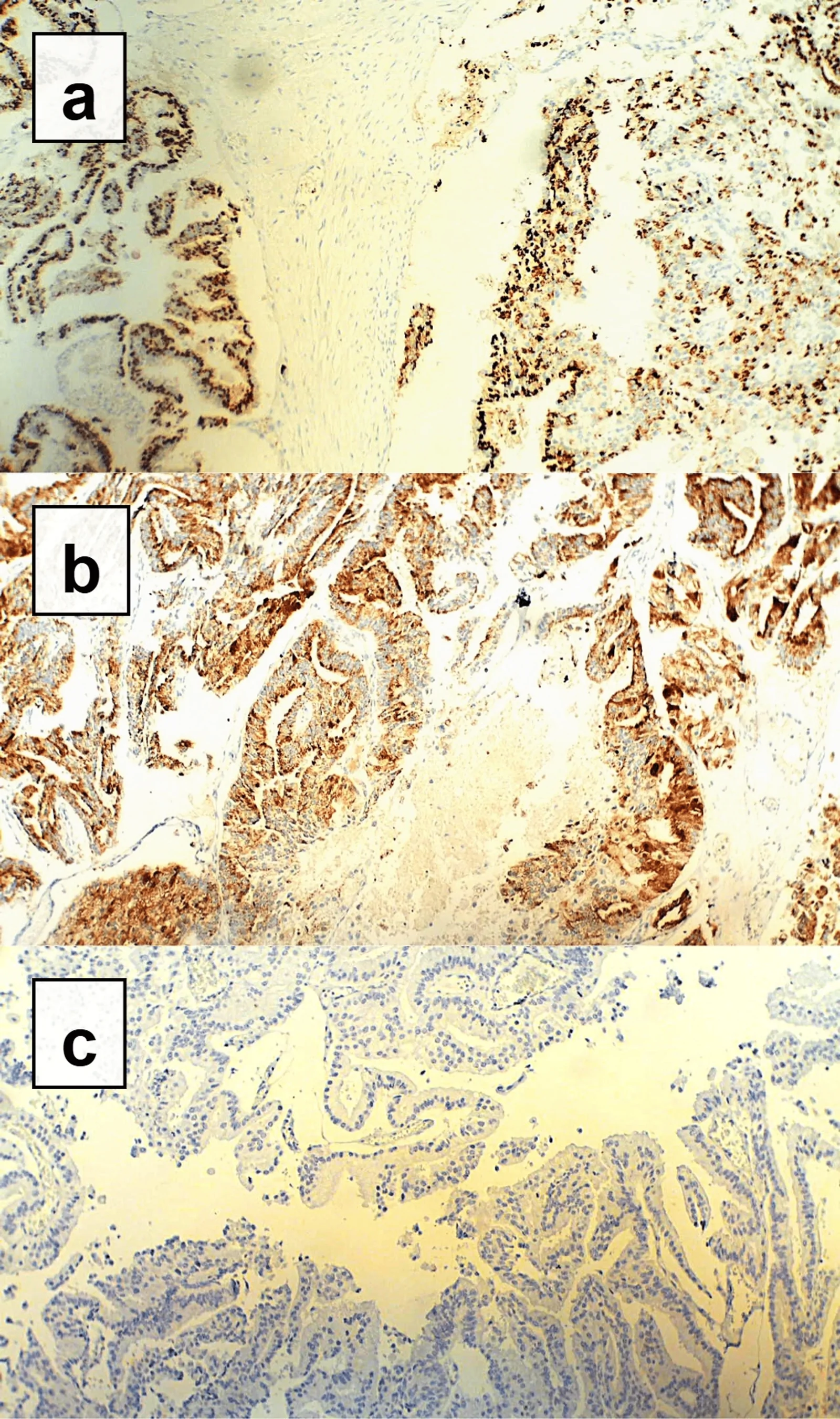
Immunohistochemical profile of the testicular metastasis. a. Prostein: Metastatic cells show positive cytoplasmic staining. b. AMACR (P504S): Strong and diffuse granular cytoplasmic staining across the metastatic proliferation. c. PSA: The metastatic cells are negative for Prostate-Specific Antigen (PSA) expression.

Medically, hormonal treatment was reinstated and intensified by the implementation of dual androgen suppression, combining ADT with ARPI (abiraterone acetate). Pathological examination of the resected specimen revealed a diffuse tumor proliferation (Figures 4-5). Immunohistochemical study showed intense and diffuse expression of prostein (P501S) and alpha-methylacyl-CoA racemase (AMACR, also known as P504S) (Figure 6). Concurrently, a total absence of expression of specific germ cell tumor markers (placental alkaline phosphatase (PLAP), octamer-binding transcription factor 3/4 (OCT3/4), AFP, and β-hCG) was noted. This profile allowed for the formal exclusion of a primary germ cell tumor hypothesis and confirmed the diagnosis of a testicular metastasis originating from prostate adenocarcinoma.

At four weeks postoperatively, early biochemical follow-up showed a favorable response with a rapid drop in the serum PSA level to 0.09 ng/mL.

## Discussion

Historically, the first case documented in the medical literature of a prostate carcinoma metastasizing to a testicle was reported by Semans in 1938 [2]. Since this index observation, the scientific literature has recorded only a very limited number of similar cases. The reference autopsy series conducted by Bubendorf et al. demonstrated that testicular metastases were present in a minuscule proportion of cases [3]. Other reviews estimate this clinical incidence at up to 4%, with the majority of these lesions being discovered incidentally during the examination of testicles resected during palliative bilateral orchiectomy in patients suffering from terminal metastatic disease [4]. The clinical presentation of a testicular metastasis as an isolated unilateral scrotal mass, in a patient with a relapse classified as oligometastatic prostate cancer (omPCa), therefore constitutes a medical event of major scientific interest [5]. The main dissemination mechanisms described to explain this atypical tropism include retrograde venous extension, arterial or lymphatic embolization, as well as direct endocanalicular propagation via the vas deferens [4].

Diagnostically, the investigation of an oligometastatic biochemical recurrence now relies on PSMA PET-CT. This next-generation imaging proves highly sensitive for detecting isolated testicular metastatic foci, even when scrotal ultrasound is falsely negative or inconclusive [6]. To avoid missing these lesions, the literature rigorously recommends including the scrotum in the standard field of view during the acquisition of PET images in these patients [6]. Histopathological analysis remains the only means of confirming the diagnosis, particularly in the elderly where the differential diagnosis primarily includes testicular lymphomas and primary germ cell tumors. However, metastases from high-grade or hormone-treated prostate adenocarcinomas may lose the expression of classic markers (PSA and prostatic acid phosphatase (PSAP)), leading to a risk of false negatives [7]. This is why the use of next-generation immunohistochemical panels is essential. Prostein (P501S) offers major diagnostic specificity, as its granular cytoplasmic expression is not inversely correlated with tumor dedifferentiation. Similarly, AMACR and the nuclear marker NKX3.1 are distinguished by extremely high sensitivity and specificity, allowing formal authentication of the prostatic origin of the mass [8-10].

The management of omPCa has recently evolved towards a multimodal paradigm. This combines metastasis-directed therapy (MDT) with systemic treatment intensification [11,12]. Orchiectomy allows for the radical ablation of the local tumor burden. In accordance with current guidelines, this intervention must be supplemented by dual androgen suppression, combining ADT with an ARPI, such as abiraterone [13]. This aggressive therapeutic approach to metachronous omPCa significantly reduces the risk of escape and achieves durable remissions with prolonged survival times [11,12].

## Conclusions

This case highlights that solitary testicular metastasis, although exceptionally rare, should be considered in the evaluation of a new scrotal mass in patients with a history of prostate cancer. These findings underscore the importance of including the scrotal region during prostate-specific membrane antigen positron emission tomography-computed tomography (PSMA PET-CT) imaging, as well as the critical role of targeted immunohistochemical markers, particularly prostein (P501S) and alpha-methylacyl-CoA racemase (AMACR), in establishing an accurate diagnosis when prostate-specific antigen (PSA) expression is inconclusive. A multimodal approach combining metastasis-directed therapy (MDT) with systemic treatment intensification using androgen deprivation therapy (ADT) and androgen receptor pathway inhibitors (ARPI) may represent a valuable strategy for managing oligometastatic recurrence in this rare clinical setting.
